# Hotspots and development frontiers of postoperative complications of AD: Bibliometric analysis – a review

**DOI:** 10.1097/MD.0000000000033160

**Published:** 2023-03-10

**Authors:** Danni Feng, Sufang Huang, Quan Wang, Xiaorong Lang, Yuchen Liu, Kexin Zhang

**Affiliations:** a Tongji Hospital, Tongji Medical College, Huazhong University of Science and Technology, Wuhan, China; b School of Nursing, Tongji Medical College, Huazhong University of Science and Technology, Wuhan, China.

**Keywords:** aortic dissection, bibliometric analysis, CiteSpace, complication, visualization, VOSviewer

## Abstract

The research on the postoperative complications of aortic dissection (AD) has received great attention from scholars all over the world, and the number of research articles in this field has consistently increased year after year. However, no bibliometric reports have been published yet to analyze the scientific output and the current situation in this field. The Bibliometrix R-package, VOSviewer, and CiteSpace software were used to conduct a bibliometric analysis of the hotspots and development frontiers of AD. A total of 1242 articles were retrieved. The USA, China, and Japan had the highest number of publications. The five keywords with the highest frequency were “analysis,” “incidence,” “acute type,” “graft,” and “risk factor.” The results also indicated that the research in related fields had shifted from surgical treatment and utilizing experience to the evidence-based exploration of risk factors and the construction of prediction models to help better manage postoperative complications of AD. This is the first bibliometric analysis of global publications on the postoperative complications of AD. The current research hotspots focus on three areas: common postoperative complications of AD, exploration of the related risk factors, and management of complications. Future research could focus on identifying risk factors through meta-analysis and using a multicenter database for AD as well as building relevant models to predict the development of complications to better facilitate the clinical management of AD patients.

## 1. Introduction

Aortic dissection (AD) have become one of the main diseases threatening the life safety of patients because of its dangerous onset, rapid progression, diverse initial symptoms, and high misdiagnosis rate. In addition, the mortality rate for AD is high, with an overall in-hospital mortality of 27.4%; the in-hospital mortality rate for type A dissections is 26% to 58%, while that for type B dissection is 11% to 31%.^[1]^ Patients who present early, untreated acute type A aortic dissection (AAAD) die at a rate of 1% to 2% per hour on the first day, and almost half of them die at the end of the first week.^[2]^ Urgent open surgical repair is the treatment of choice for AAAD, whereas type B dissection is usually treated with medical therapy, thoracic endovascular aortic repair (TEVAR), or surgery depending on the severity of the disease.^[3,4]^ In recent years, new studies have indicated that the overall mortality for AD decreased in Australia, the United States, and many European countries between 2000 and 2017.^[5]^ However, despite improved diagnostic and therapeutic techniques, postoperative complications remain an independent risk factor for in-hospital mortality.^[6]^ As type A dissections involve the ascending aorta, aortic arch, and descending aorta, surgery is often complex and has a high surgical risk.^[7]^ It is prone to complications such as hypoxemia,^[8]^ acute respiratory distress syndrome,^[9]^ delirium,^[10]^ renal insufficiency,^[11]^ cardiac insufficiency,^[12]^ impaired neurological function,^[13]^ and paraplegia.^[14]^ Type B dissections are often treated using TEVAR. Although less invasive and having a shorter time frame, postoperative complications such as endoleaks,^[15]^ secondary type A dissection,^[16]^ stroke,^[17]^ and paraplegia^[18]^ can still occur. Further, AD results in different complications for different patient groups, such as the elderly, children, and pregnant women.^[19–23]^ Recently, the research on the postoperative complications of AD has received considerable attention from scholars worldwide, and the number of research articles in this field has increased annually. However, no bibliometric reports have been published yet to analyze the scientific output and the current situation in this field. Therefore, it is important to adopt visual methods to illustrate the global status, research hotspots, and development frontiers of the postoperative complications of AD. Using the Bibliometrix R-package, VOSviewer, and CiteSpace software, we conducted a bibliometric analysis of relevant studies on the postoperative complications of AD and provide a new perspective for future research to improve the clinical management, prognosis, and rehabilitation of patients who present with AD.

## 2. Methods

### 2.1. Data source and search strategy

A bibliometric literature search was performed online using the Web of Science Core Collection (WoSCC) on May 27, 2022, with the time span for publication set to 1991 to 2022. Three Medical Subject Headings terms were used for the search. Term A was “aortic dissection,” Term B was “complication,” and Term C was “postoperative.” The search criteria for the included publications were the following: TS = (aortic dissection) AND TS = (complication) AND TS = (postoperative). Among the types of documents included were original research articles and reviews (including meta-analysis) written in English. The exclusion criteria were as follows: repeated published literature; and non-English literature. A total of 1242 articles were included in the study, and the literature was exported and saved. All records and references were formatted and stored as plain text files in.txt format.

### 2.2. Data analysis

Visual analysis was performed using the Bibliometrix R-package, VOSviewer, and CiteSpace software. The R tool Bibliometrix (Version 3.2.1) of R-Studio (Version 4.1.0) was used to carry out a comprehensive bibliometric analysis, combined with the web-based tool Biblioshiny, to export and manage the data from WoSCC.^[24]^ Its basic functions include an analysis of the main information, average number of citations per year, number of papers published per year, journal sources, frequently cited journals, high-yield authors, high-yield institutions, national number of papers published, author cooperation network, and world cooperation map. VOSviewer is a bibliometric analysis software used to build and view bibliometric maps.^[25]^ It is based on the principle of collaborative and co-occurrence data and can be used to draw scientific maps in various domains of knowledge. Finally, CiteSpace is an information visualization software developed using Java.^[26]^ It mainly evaluates the literature in a specific field based on co-citation analysis theory and the path-finder algorithm to explore the key paths and knowledge inflection point for the evolution of the considered field and formulate an analysis of the potential dynamic mechanism of the discipline’s evolution and the exploration of the frontiers of the discipline’s evolution through the drawing of a series of visualization maps.

## 3. Results

### 3.1. Basic characteristics of publications

#### 1.3.1. Main information of the published papers.

Of the 1339 articles retrieved from the search, 97 were excluded after filtering based on the exclusion criteria. Thus, 1242 articles published in 288 sources (journals, books, etc.) were considered. The publication dates of the articles ranged from 1991 to 2022. Among the articles, the average number of years since publication was 9.95, the average number of citations per article was 22.23, the average number of citations per year per article was 1.714, and the number of keywords, author’s keywords, authors, and coauthors per article were 1866, 1940, 5451, and 6.03, respectively. Finally, the collaboration index was 4.44. All data are shown in Table 1.

**Table 1:** Main information about data of all published papers related to research on complications after aortic dissection.

Description	Results
Timespan	1991:2022
Sources (journals, books, etc)	288
Documents	1242
Average years from publication	9.95
Average citations per documents	22.23
Average citations per year per doc	1.714
References	18158
Document contents	
Keywords plus (ID)	1866
Author’s keywords (DE)	1940
Authors	
Authors	5451
Author appearances	7488
Authors of single-authored documents	19
Authors of multi-authored documents	5432
Authors collaboration	
Single-authored documents	19
Documents per author	0.228
Authors per document	4.39
Co-authors per documents	6.03
Collaboration Index	4.44

#### 2.3.1. Annual quantity and average number of annual citations of published papers.

**Figure 1.**
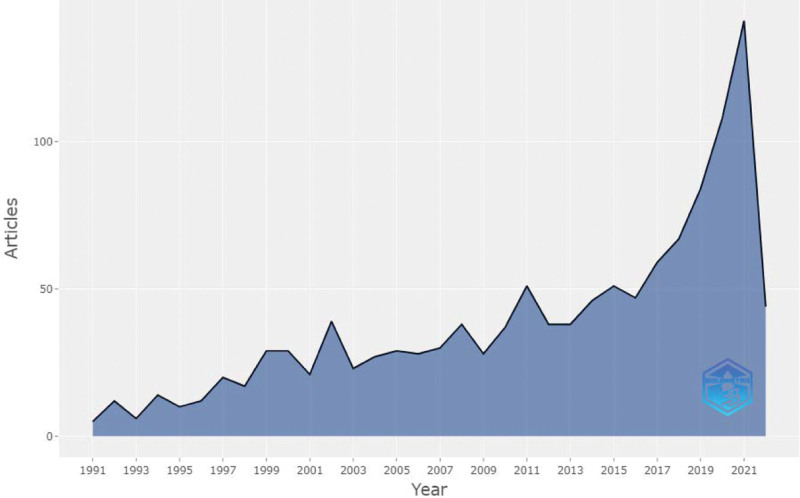
Annual number of published papers.

**Figure 2.**
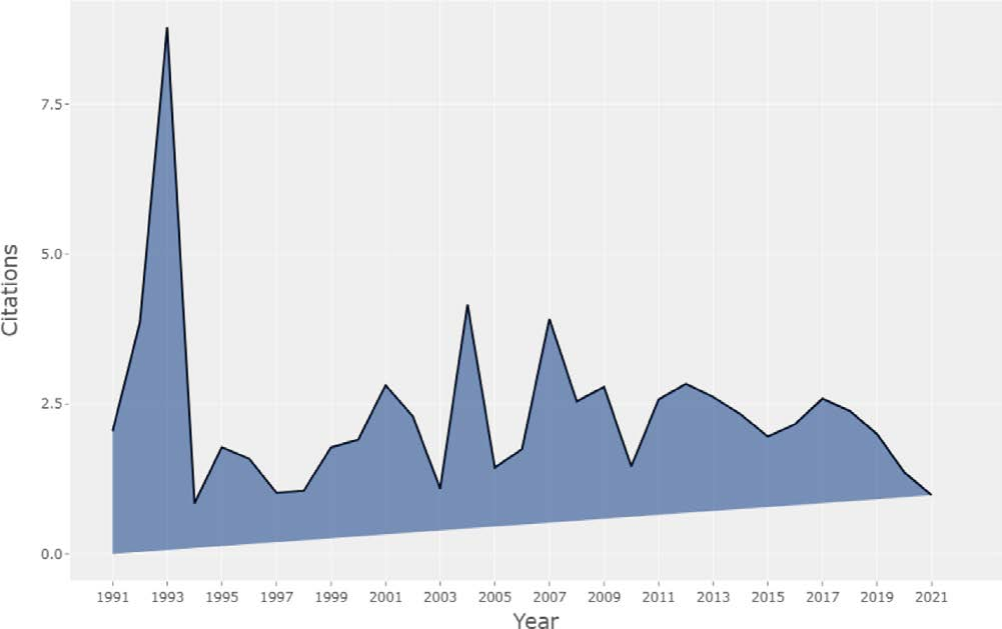
Average annual citations of published papers.

In Figure 1, the Bibliometrix package (version 3.2.1) was used to illustrate the distribution trend of the annual number of published papers. Considering the number of published papers, it can be stated that the academic research conducted in this field from 1991 to 2003 was in its initial stage. Since then, it has grown annually, increasing rapidly from 2016 to 2021, and peaked in 2021 (141 papers), with an annual growth rate of 7.27%. As the articles in 2022 were not included, there was a downward trend. The average number of articles cited per year fluctuates, with the highest average number of citations for each paper reaching 8.8 times per year in 1993 (see Fig. 2). Upon closer inspection, the peak in 1993 was attributed to a paper published by Svensson.^[27]^ This study explored the factors that influenced early death and postoperative complications through a retrospective analysis of patients who underwent thoracoabdominal aortic operations between 1960 and 1991; it received a total of 889 citations.

#### 3.3.1. Features of publication papers.

The documents were published from 288 publication sources, which were ranked by the h-index. The top ten published papers with the highest h-index values are listed in Table 2. We used five basic indicators to define the quantitative characteristics of these sources: h-index, impact factor (IF), total citations of publications, number of publications, and year in which each publication started to publish the articles relevant to the field (PY_start).

**Table 2:** Top 10 publication sources.

Element	h_index	IF (2020)	TC	NP	PY_start
ANNALS OF THORACIC SURGERY	37	4.330	3866	97	1992
JOURNAL OF VASCULAR SURGERY	35	4.268	5001	95	1991
EUROPEAN JOURNAL OF CARDIO-THORACIC SURGERY	30	4.191	2453	80	1991
JOURNAL OF THORACIC AND CARDIOVASCULAR SURGERY	28	5.209	3208	62	1992
ANNALS OF VASCULAR SURGERY	13	1.466	550	46	2001
EUROPEAN JOURNAL OF VASCULAR AND ENDOVASCULAR SURGERY	13	7.069	572	15	2000
JOURNAL OF CARDIAC SURGERY	13	1.620	590	37	1992
JOURNAL OF CARDIOVASCULAR SURGERY	11	1.888	438	39	1992
JOURNAL OF ENDOVASCULAR THERAPY	11	3.487	421	18	2002
GYNECOLOGIC ONCOLOGY	10	5.482	769	13	1996

IF = impact factor, NP = number of publications, TC = total citations.

As shown in Table 2, if the h-index value is considered, the papers that were published in the Annals of Thoracic Surgery had the largest influence on the research on the postoperative complications of AD published from January 1992 to November 2021, with a total of 37 articles. In addition, it published 97 papers in this research field overall and ranked first among all the journals. The total number of citations of the Journal of Vascular Surgery was 5001, which was the highest. If the IF is considered, the European Journal of Vascular and Endovascular Surgery had an IF of 7.069, ranking first among all the journals, followed by Gynecologic Oncology (5.482) and the Journal of Thoracic and Cardiovascular Surgery (5.209).

### 3.2. Cooperation, active authors, and institutions

#### 1.3.2. Country/region distribution of publications.

**Figure 3.**
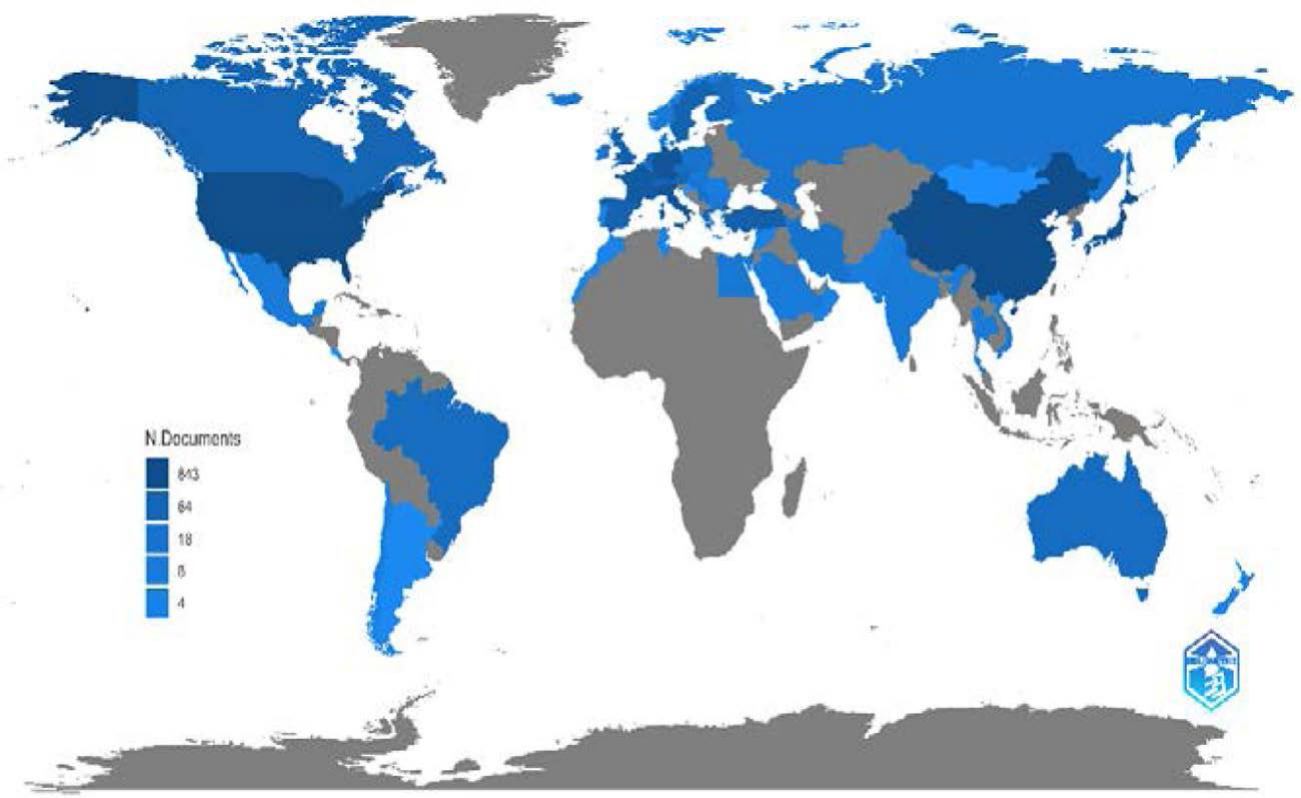
Country-/region-based distribution of published papers.

Figure 3 shows the number of published papers for each country. The publications were distributed across 56 countries/regions. The darker the color of the country/region, the higher the number of articles published in that country or region. As shown in Figure 3, the USA, China, and Japan had the most publications.

#### 2.3.2. Countries/regions network.

**Figure 4.**
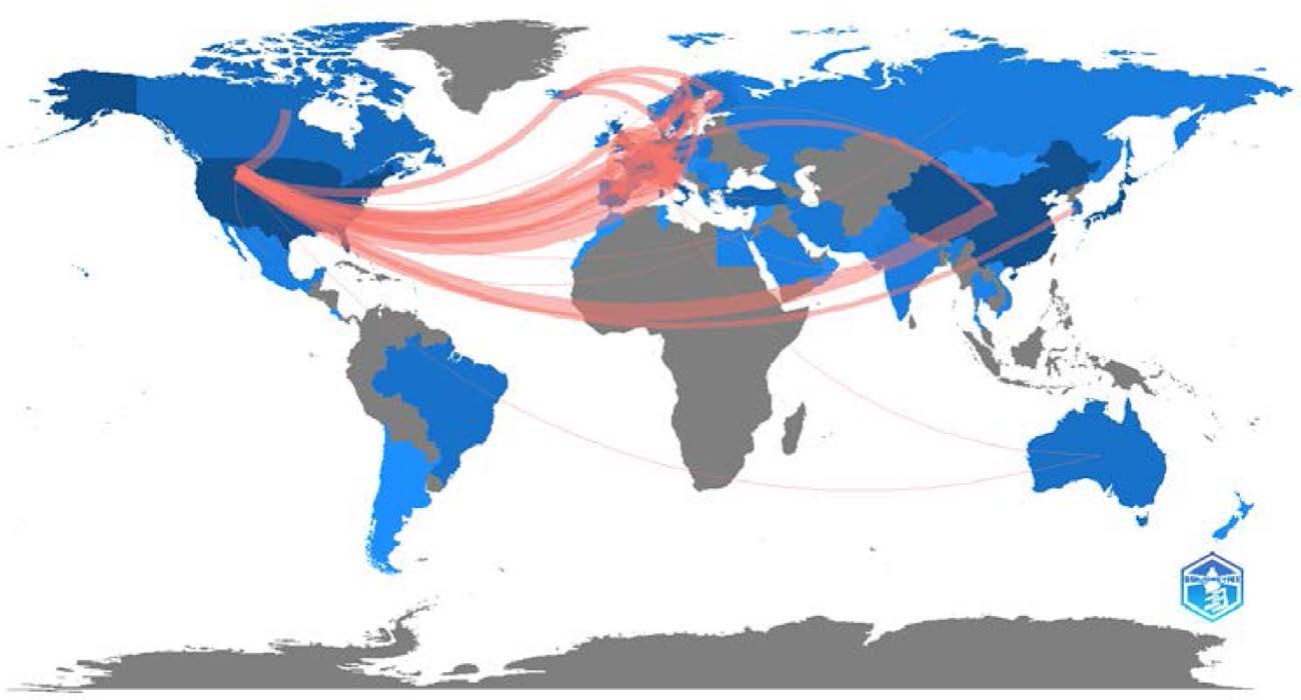
Country collaboration map of published papers.

Figure 4 shows the cooperative relationships among countries/regions. From Figure 4 and Table 3, we can see that Germany, Italy, the USA, France, and the United Kingdom cooperate closely.

**Table 3:** The cooperation among countries/regions.

From	To	Frequency
GERMANY	ITALY	12
USA	CHINA	12
USA	ITALY	11
ITALY	FRANCE	10
GERMANY	UNITED KINGDOM	9
ITALY	AUSTRIA	8
ITALY	UNITED KINGDOM	8
USA	GERMANY	8
GERMANY	AUSTRIA	7
ITALY	SPAIN	7

#### 3.3.2. Active authors and distribution of institutions.

**Figure 5.**
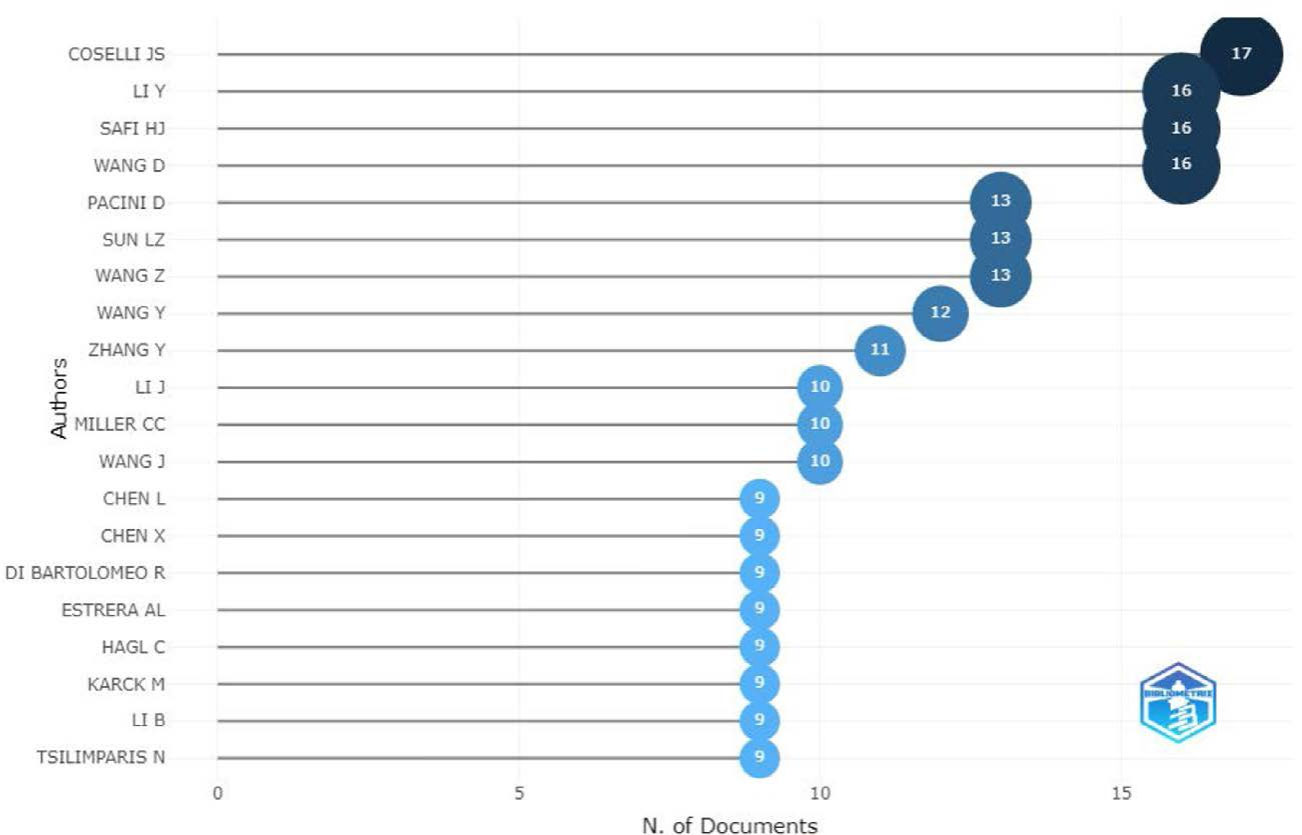
Top 20 most relevant authors from all published papers.

**Figure 6.**
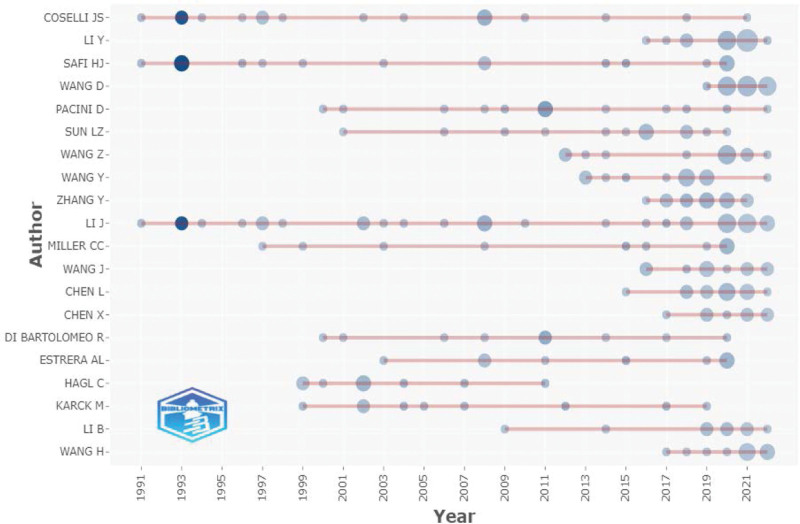
Top 20 authors’ published papers over time.

**Figure 7.**
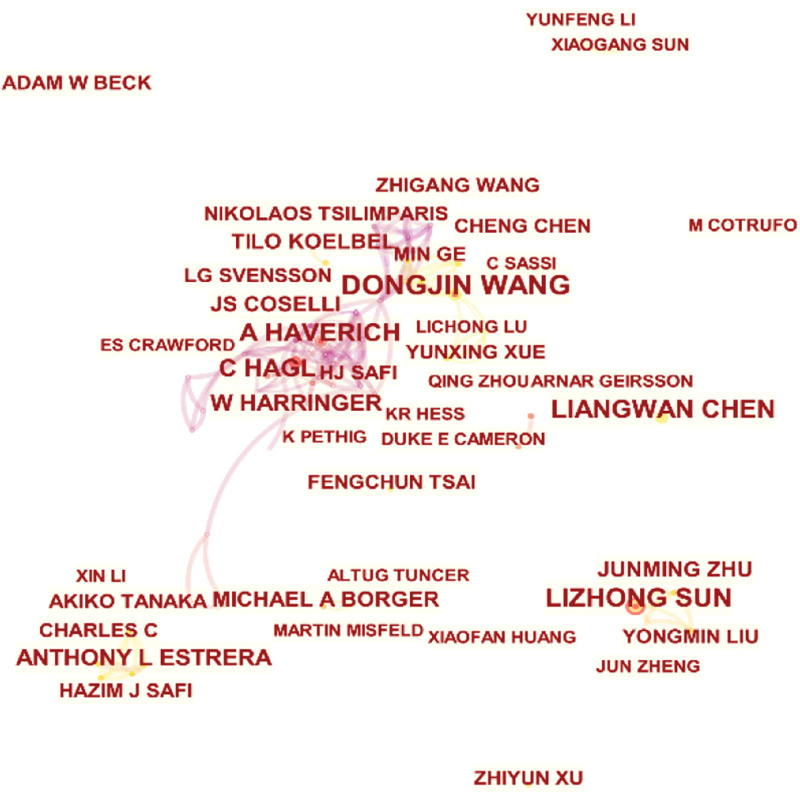
Co-occurrence knowledge map of research authors.

**Figure 8.**
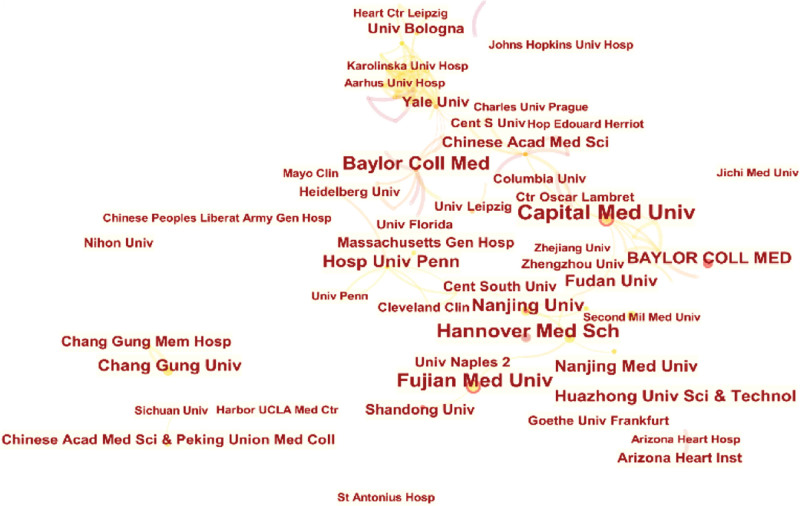
Co-occurrence knowledge map of research institutions.

Figure 5 shows the top 20 most relevant authors among all the published papers. The top five authors were J. S. Coselli, Y. Li, H. J. Safi, D. Wang, and D. Pacini (reference numbers 17, 16, 16, 16, and 13, respectively). Figure 6 shows the top 20 authors’ published papers over time. Coselli had the highest number of publications (17) during the considered period. His first publication on the subject was in 1991, and his greatest scientific production was registered in 2008. Next, Figure 7 shows the co-authorship network among the authors. The network map had 946 nodes and 1262 connections, and the network density was 0.0028; this indicates that the researchers in this field did not cooperate closely, and only a small number of academic teams were formed. In the field of research on the postoperative complications of AD, the top three research institutions according to the total number of papers published were Capital Medical University, Fujian Medical University, and Fudan University (Table 4). Further, Figure 8 shows the co-occurrence knowledge map of the research institutions drawn by CiteSpace with a total of 621 nodes. However, there were only 469 connections between the nodes, and the distribution among institutions was relatively scattered, indicating that the research on the postoperative complications of AD lacks close cooperation as a whole.

**Table 4:** Top 10 most relevant affiliations.

Affiliations	Articles
CAPITAL MED UNIV	62
FUJIAN MED UNIV	49
FUDAN UNIV	40
BAYLOR COLL MED	39
CHANG GUNG UNIV	29
HANNOVER MED SCH	29
HUAZHONG UNIV SCI AND TECHNOL	29
UNIV FLORIDA	27
UNIV BOLOGNA	26

### 3.3. Frequently cited documents

The number of citations in a publication indicates the popularity of a particular study. Table 5 lists the top 10 globally cited published papers related to research on the postoperative complications of AD, which were ranked based on globally cited articles. Table 6 lists the top 10 locally cited published papers ranked according to the locally cited articles. The top publication was the paper by Svensson (1993), with 889 citations, and its local citations were 29. Most publications with high citation counts were distributed between 1992 and 2008.

**Table 5:** Top 10 global cited published papers.

Documents (Author, Year, Source)	Total citations	TC per year	Normalized TC
SVENSSON LG, 1993, J VASC SURG	889	29.63	3.49
DAVID TE, 1992, J THORAC CARDIOVASC SURG	829	26.74	7.16
MURKIN JM, 2007, ANESTH ANALG	515	32.19	8.76
SANO T, 2004, J CLIN ONCOL	496	26.11	6.63
SVENSSON LG, 1993, J THORAC CARDIOVASC SURG	477	15.90	1.87
LEURS LJ, 2004, J VASC SURG	420	22.11	5.61
CAMBRIA RP, 2002, ANN SURG	268	12.76	5.85
BERNARD Y, 2001, AM J CARDIOL	234	10.64	3.96
FINKBOHNER R, 1995, CIRCULATION	214	7.64	4.46
XENOS ES, 2008, J VASC SURG	211	14.07	5.92

TC = total citations.

**Table 6:** Top 10 local cited published papers.

Documents (Author, Year, Source)	Local citations	Global citations	LC/GC ratio (%)	Normalized local citations	Normalized global citations
SVENSSON LG, 1993, J VASC SURG	29	889	3.26	4.58	3.49
EHRLICH MP, 2000, CIRCULATION	24	169	14.20	11.23	4.03
EHRLICH M, 1998, CIRCULATION	17	94	18.09	7.81	3.72
KAZUI T, 1992, ANN THORAC SURG	16	140	11.43	5.19	1.21
LEURS LJ, 2004, J VASC SURG	16	420	3.81	6.55	5.61
FUSCO DS, 2004, ANN THORAC SURG	15	108	13.89	6.14	1.44
VASILEV SA, 1996, GYNECOL ONCOL	14	84	16.67	7.64	2.03
GAUL C, 2007, STROKE	13	158	8.23	10.00	2.69
MISFELD M, 2012, ANN THORAC SURG	13	127	10.24	22.45	4.48
MOIZUMI Y, 2005, ANN THORAC SURG	11	79	13.92	10.63	3.23

### 3.4. Keyword analysis

#### 1.3.4. Co-occurrence analysis.

**Figure 9.**
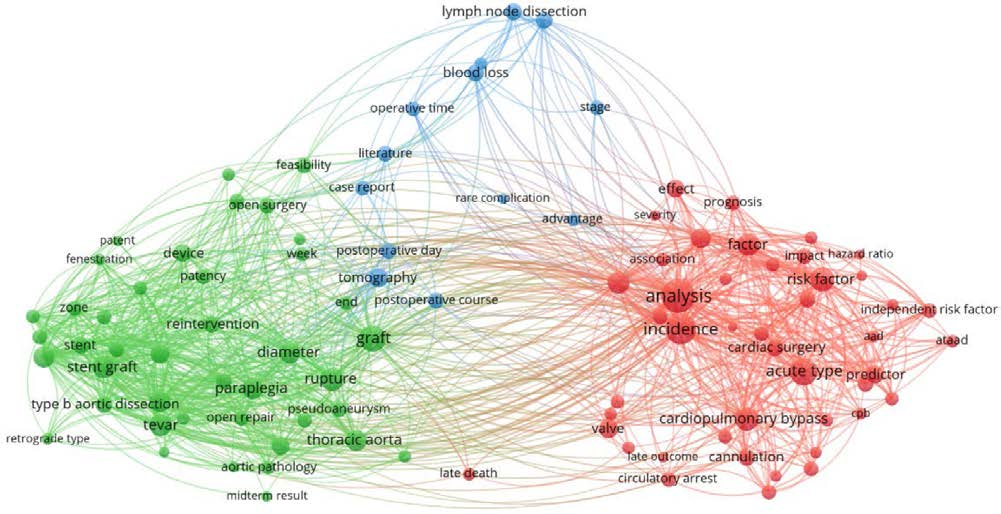
Co-occurrence network of high-frequency keywords in research on complications after aortic dissection.

As shown in Figure 9, VOSviewer (version 1.6.18) was used to the conduct co-occurrence analysis of the keywords. A total of 23,454 keywords were extracted from the 1242 papers. We limited the number of occurrences to 25 or more and selected the top 100 keywords with 3901 co-occurrence links distributed in five clusters. Table 7 summarizes the top 20 high-frequency keywords in the research on the postoperative complications of AD. The top five keywords with the highest frequency were “analysis,” “incidence,” “acute type,” “graft,” and “risk factor.”

**Table 7:** Top 20 high-frequency keywords.

Ranking	Term	Occurrences	Relevance score
1	analysis	300	0.550
2	incidence	234	0.551
3	acute type	191	1.243
4	graft	184	0.418
5	risk factor	156	1.273
6	rupture	137	0.440
7	cardiopulmonary bypass	134	1.052
8	difference	132	0.417
9	stent graft	129	1.210
10	TEVAR	128	0.846
11	factor	125	0.834
12	diameter	120	0.518
13	paraplegia	120	0.602
14	endoleak	116	1.585
15	thoracic endovascular aortic repair	115	0.909
16	thoracic aorta	113	0.419
17	significant difference	112	0.795
18	valve	98	0.592
19	tomography	93	0.324
20	endovascular repair	91	1.064

TEVAR = thoracic endovascular aortic repair.

#### 2.3.4. Keyword clustering analysis.

**Figure 10.**
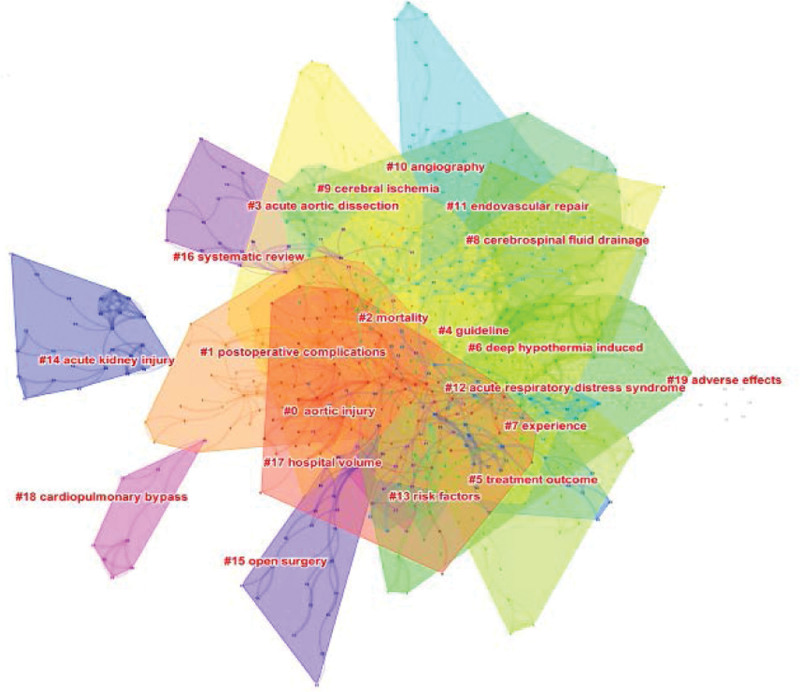
Keyword clustering network in research on complications after aortic dissection.

Keyword clustering analysis is a process based on keyword co-occurrence analysis that simplifies the keyword co-occurrence network relationship into a relatively small number of clusters using clustering statistics. This is performed by running CiteSpace, checking the “Purning” option, and selecting the log-likelihood ratio algorithm to determine the research frontier. In the literature, 683 hot spots, 1321 links, and a keyword clustering network map with a density of 0.057 were obtained, as shown in Figure 10. The modularity Q score of the clustering map was 0.7845, and the weighted mean silhouette value was 0.9075. The cluster structure is significant and reasonable. The largest cluster (Cluster #0) out of the 20 clusters was associated with “aortic injury,” followed by “postoperative complications” (Cluster #1), “mortality” (Cluster #2), and “acute aortic dissection” (Cluster #3).

#### 3.3.4. Keywords burst detection analysis.

**Figure 11.**
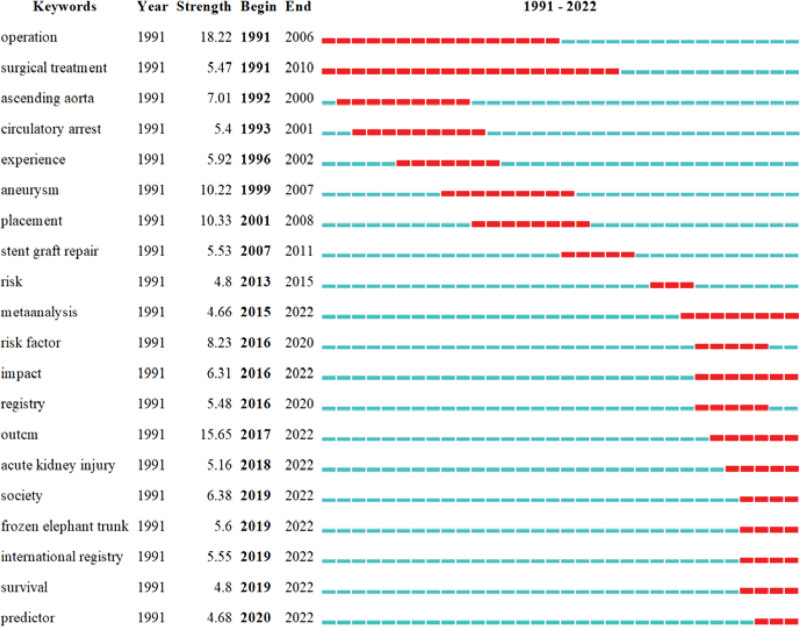
Top 20 keywords with the strongest citation bursts.

Burst words are keywords that are cited more frequently during a certain period of time and can be used to reflect research trends within that period of time. This is carried out by running CiteSpace and selecting the parameter “Burst Terms.” Figure 11 shows that research in related fields has shifted from surgical treatment and utilizing experience to the evidence-based exploration of risk factors and the construction of prediction models to help better manage the related postoperative complications of AD.

## 4. Discussion

### 4.1. Global research trends for the postoperative complications of AD

In this study, the Bibliometrix R-package, VOSviewer, and CiteSpace software were used to conduct a bibliometric analysis of the hotspots and development frontiers of the research on the postoperative complications of AD based on the WoSCC. A total of 1242 original articles and published reviews from 1991 to 2022 were considered. The results showed that the number of annual publications increased during this period, indicating that the postoperative complications of AD have received increasing attention, especially from 2016 to 2021, peaking in 2021. The USA published the most articles, followed by China, Japan, Germany, and Italy. The top three research institutions in terms of the number of papers published were Capital Medical University, Fujian Medical University, and Fudan University. J. S. Coselli (17 publications) was identified as the most active author in the studied research field, followed by Y. Li (16 publications). Coselli primarily explained the common postoperative complications and their influencing factors for patients with dissections through retrospective research,^[27–30]^ proposed measures to effectively control the complications from the management level to prolong survival time,^[31]^ prospectively studied the predictive factors of postoperative respiratory failure,^[32]^ and explored the postoperative outcomes in elderly patients who presented with AD.^[33]^ However, cooperation in this field of research is not high among both researchers and research institutions. Therefore, team cooperation and academic exchanges across disciplines, institutions, and regions should be strengthened in the future. In 1993, the average number of citations for each paper was the highest, growing 8.8 times per year, which laid the foundation for subsequent research.

### 4.2. Research hotspots

The co-occurrence network of high-frequency keywords presented in Figure 9 shows that the research on the postoperative complications of AD can be divided into three clusters. Based on the frequency and link strength of keywords in the different areas, we summarized the following research hotspots for the postoperative complications of AD:

#### 1.4.2. Common complications.

Researchers have explored the common postoperative complications of AD, mainly through case reports, retrospective studies, and prospective studies. At present, nervous system-related complications, such as limb ischemia, hemiplegia, and paraplegia, are the most frequently reported complications. Spinal cord ischemia and subsequent paraplegia or direct paraplegia are rare, and catastrophic complications occur during the postoperative treatment of patients who present with acute AD, which seriously affects the postoperative rehabilitation of such patients and significantly increases operative mortality.^[34]^ In 1997, Sakurada reported the case of a 52-year-old female patient with DeBakey type I acute AD who developed paraplegia after emergency surgery.^[35]^ Thereafter, many researchers have reported paraplegia in patients with AD.^[36–39]^ In addition, some studies have reported cases of internal leakage,^[40,41]^ secondary dissection,^[42]^ and hypoxemia^[43]^ post AD. Svensson was the first to perform a retrospective analysis of 1509 patients who underwent thoracoabdominal aortic repair between 1960 and 1991.^[27]^ The results showed that the overall incidence of paraplegia or paraparesis was 16% (234/1509), and kidney failure (posterior creativity level > 3 mg/dL or diagnosis) occurred in 18% (269/1509) of the patients. Coselli^[28]^ also retrospectively analyzed 372 patients with thoracoabdominal aneurysms, 25% (93/372) of whom had AD, to understand the incidence of renal failure and postoperative neurological impairment. An increasing number of researchers have understood the postoperative complications of AD through retrospective studies.^[44–49]^ Relatively few prospective studies have focused on the risk factors associated with the postoperative complications of AD^[32,50]^ and the implementation effect of the new protocol.^[51,52]^ Chen et al^[53]^ conducted a prospective study on 62 patients with type B aortic dissection from 1999 to 2005 to explore the postoperative clinical results of patients at different stages of the disease. Williams used a detailed prospective clinical database to explore the incidence and outcome of the complications of retrograde ascending AD in patients who underwent TEVAR between 2005 and 2010.^[54]^

#### 2.4.2. Risk factors.

Exploring the effect of the risk factors for the early recognition of complications and the improvement of patient prognosis is crucial. Many researchers have explored the factors that influence the postoperative complications of AD through retrospective cohort studies. Khoynezhad et al^[55]^ retrospectively analyzed 153 patients who underwent 184 TEVARs between 1998 and 2005 to explore the risk factors associated with the neurologic deficits that occur after TEVAR. The incidence of stroke and spinal cord injury after TEVAR was 4.3% (8/184). Further, the risk factors associated with stroke are obesity, intraoperative blood loss, and vascular embolism. Aneurysms as an underlying pathology, the use of an iliac conduit, and the coverage of the hypogastric artery were all associated with spinal cord injury. Liu et al^[56]^ explored the risk for delirium after type A AD in 100 patients who underwent Sun’s procedure between 2014 and 2016 through a retrospective study. The results showed that cerebrovascular history, duration of surgery and cardiopulmonary bypass, postoperative hypoxia, and intubation time were independently associated with the development of delirium. Svensson conducted a prospective study with 98 patients who underwent thoracoabdominal aortic aneurysm repair (35% of patients presented with AD) to explore the independent predictors of respiratory failure. The results showed that chronic pulmonary disease and cardiac and renal complications are independent predictors of respiratory failure. In patients with chronic pulmonary disease, the only independent predictor was FEF_25_.^[32]^ Conzelmann et al^[50]^ conducted a multi-center prospective study on 2137 patients with AAAD using the German Registry for Acute Aortic Dissection Type A from 2006 to 2010 to explore the risk factors for neurological dysfunction, and the study found that new postoperative neurological dysfunction was associated with extensive malperfusion syndrome, supraaortic vessel dissection, and operative time. In addition, some scholars have conducted research on the unique traumatic stress caused by surgery, such as post-traumatic stress disorder (PTSD). Lin conducted a prospective cohort study with 224 patients who presented with AAAD between 2017 and 2019 to explore the incidence and risk factors of PTSD. The incidence of PTSD was 21.4%. Depressive symptoms and women are risk factors related to PTSD, while optimism is a protective factor in patients who present with AAAD.^[57]^ In recent years, an increasing number of researchers have devoted themselves to exploring the risk factors for the postoperative complications of AD, such as delirium,^[56,58]^ acute respiratory distress syndrome,^[59]^ hypoxemia,^[60]^ distal segment aortic enlargement,^[61]^ distal stent graft-induced new entry,^[62]^ acute kidney injury,^[63,64]^ and hepatic dysfunction.^[65]^

#### 3.4.2. Management of complications.

There are many kinds of postoperative complications of AD that affect patients’ rehabilitation, prognosis, and quality of life after discharge and even endanger their lives. Therefore, the effective identification and management of complications is a problem that has been explored by researchers. De Santo et al^[66]^ were the first to evaluate the effect of continuous pulmonary perfusion during retrograde cerebral perfusion in a prospective series of 22 AAAD patients. The results showed that continuous pulmonary perfusion had a positive effect on postoperative pulmonary function, which was supported by the enhanced preservation of postoperative pulmonary gas exchange (PaO_2_/FiO_2_ ratio) and reduction of ventilator support. Roseborough et al^[67]^ reported a case of acute cerebral hypoperfusion in a 66-year-old patient with AAAD postoperatively. They reported for the first time that an ischemic stroke caused by the malperfusion of arch vessels was successfully reversed by stenting of the involved arch vessels. The patients were followed up with, and their neurological examinations were normal and without abnormal perfusion. Fleck et al^[68]^ described a case report study and showed that cerebrospinal fluid drainage was an effective treatment for paraplegia after stent graft implantation for acute type B aortic dissection, which was also proven by subsequent studies.^[69,70]^ A systematic review and meta-analysis published by Zhang et al^[71]^ on the prevention of spinal cord ischemia through prophylactic cerebrospinal fluid drainage after TEVAR demonstrated that prophylactic cerebrospinal fluid drainage can effectively prevent spinal cord ischemia in patients after AD. Li et al^[72]^ proposed a novel simplified thrombo-inflammatory prognostic score to predict in-hospital complications in patients with AAAD, which has great clinical significance for identifying high-risk patients. In addition, Liu et al^[73]^ developed a nomogram combined with metabolic acidosis to predict high-risk acute type B aortic dissection patients with organ hypoperfusion after TEVAR. Recently, many scholars have committed to developing new technologies to prevent or minimize the harm caused by complications^[74]^ and, through the establishment of models or specific algorithms, to identify and predict the occurrence of complications early so as to better realize individualized risk assessment and perioperative management.^[75–79]^

### 4.3. Research frontiers

The fact that the city burst time of the keywords in Figure 11, such as “meta-analysis,” “risk factor,” “impact,” “international registry,” “survival,” and “predictor,” has continued till 2022 and is still ongoing demonstrates that these directions have great potential. In recent years, the research focus has shifted from surgical treatment and experience utilization to the evidence-based exploration of risk factors and the construction of prediction models. Buth et al^[80]^ prospectively enrolled 606 patients (215 with AD) using the European Collaborators on Stent/Graft Techniques for Aortic Aneurysm Repair database to assess the incidence and risk factors for paraplegia or paraparesis and intracranial stroke during the perioperative period. Eggebrecht et al^[81]^ explored the incidence and patient outcomes of retrograde ascending AD after TEVAR using data from 28 centers participating in the European Registry on Endovascular Aortic Repair Complications from 1995 to 2008. Jakob et al explored the incidence of postoperative complications and clinical outcomes for patients according to the International E-Vita Open Registry.^[82,83]^ However, in these studies, the patients with AD were only explored as a sub-category of the research objective. In 2012, Conzelmann et al^[50]^ carried out a multi-center prospective study based on the German Registry for Acute Aortic Dissection Type A to explore the risk factors for neurological dysfunction during the perioperative period of AAAD. Scholars have subsequently used the International Registry of Acute Aortic Dissection^[84,85]^ and the Nordic Consortium for Acute Type A Aortic Dissection^[86,87]^ to understand the incidence of the postoperative complications of AD and its impact on prognosis. In addition, an increasing number of researchers have carried out meta-analysis to understand the current postoperative complications of AD^[88–90]^ and, through retrospective or prospective research, to comprehend the risk factors of complications^[61,63,64]^ to predict the occurrence of complications, which has laid the foundation for the enhanced prognosis of patients with AD, the improved quality of life, and the provision of better clinical management.

## 5. Conclusions

In this study, we used the Bibliometrix R-package, VOSviewer, and CiteSpace software to visually analyze 1242 studies on the postoperative complications of AD published between 1991 and 2022, which includes the publication trend, publication source, core author group, country of publication, high-yield institutions, frequently cited articles, and keyword analysis of related studies, to identify the current research hotspots and development trends in this field. This is also the first bibliometric analysis of global publications on the postoperative complications of AD. The results show that the number of articles in this field is increasing annually, indicating that an increasing number of researchers are paying attention to this field. The current research hotspots focus on three areas: common postoperative complications of AD, exploration of the related risk factors, and management of complications. Future research could focus on identifying risk factors through meta-analysis and using a multicenter database for AD as well as on building relevant models to predict the development of complications to facilitate the clinical management of AD patients. In addition, there is still a lack of cooperation between countries. In the future, we should continue to carry out interdisciplinary, inter-institutional, and cross-regional team cooperation and academic exchanges and publish more influential research to better help promote patients’ postoperative rehabilitation and improve their quality of life after discharge.

## 6. Limitations

The potential limitation of our study lies in the fact that the publications related to the postoperative complications of AD were only extracted from the WoSCC database and Scopus, PubMed, Medline, Embase, and other databases were not considered; therefore, the data in the present study might not be comprehensive. In addition, we only selected the articles that were published in English, which resulted in a language bias.

## Author contributions

**Formal analysis:** Danni Feng.

**Funding acquisition:** Danni Feng.

**Methodology:** Xiaorong Lang.

**Software:** Quan Wang.

**Supervision:** Sufang Huang, Quan Wang, Kexin Zhang, Yuchen Liu.

**Validation:** Sufang Huang.

**Visualization:** Sufang Huang.

**Writing – original draft:** Danni Feng.

**Writing – review & editing:** Danni Feng.
